# UFL1 Alleviates Lipopolysaccharide-Induced Cell Damage and Inflammation via Regulation of the TLR4/NF-*κ*B Pathway in Bovine Mammary Epithelial Cells

**DOI:** 10.1155/2019/6505373

**Published:** 2019-02-10

**Authors:** Chengmin Li, Lian Li, Kunlin Chen, Yiru Wang, Fangxiao Yang, Genlin Wang

**Affiliations:** College of Animal Science and Technology, Nanjing Agricultural University, Nanjing 210095, China

## Abstract

In recent studies, UFL1 (ubiquitin-like modifier 1 ligating enzyme 1) has been identified as a significant regulator of NF-*κ*B signaling and cellular stress response, yet its physiological function in LPS-stimulated bovine mammary epithelial cells (BMECs) remains unknown. In this study, we investigated the modulating effect of UFL1 on the regulation of LPS-induced inflammation and cell damage, with a focus on apoptosis, ER stress, autophagy, oxidative stress, and the TLR4/NF-*κ*B signaling pathway. The results showed that UFL1 depletion aggravated the LPS-induced inflammatory response and cell damage by positively regulating the TLR4/NF-*κ*B pathway (increased the expression of TLR4, NF-*κ*B P65 in nuclear, and phospho-I*κ*B*α*), exacerbating LPS-induced ER stress (increased the expression of CHOP, Hsp70, and GRP78), apoptosis (increased the expression of Bax/Bcl-2 and activity of caspase-3), autophagy (increased LC3-II and decreased P62 expression), and oxidative stress (decreased SOD and CAT levels and increased MDA levels). Overexpression of UFL1 suppressed the activation of the TLR4/NF-*κ*B pathway and relieved the LPS-induced ER stress, apoptosis, autophagy, and oxidative stress, thereby alleviating the inflammatory response and cell damage. Collectively, UFL1 may play an important role during the inflammatory response and thereby acts as a potential therapeutic target for bovine mastitis.

## 1. Introduction

Mastitis is an inflammation of the mammary gland, which is most often caused by the infection of mammary gland by various microorganisms, including gram-negative bacteria [1]. Lipopolysaccharide (LPS) released from gram-negative bacteria is considered to be an important stimulus of bovine mammary epithelial cell inflammation [2]. Mammary epithelium, the predominant cell type in the mammary gland, can bind LPS to pathogen-associated molecular pattern molecules via activating various pattern recognition receptors (PRRs), e.g., toll-like receptors (TLRs) and nucleotide oligomerization domain-like receptors (NLRs) [3–5]. Generally, the binding of LPS to a TLRs or NLRs can lead to a sequential transcriptional regulatory cascades, resulting in inflammation. Nuclear factor-kappa B (NF-*κ*B), which acts as a key regulator involved in the inflammation associated with the development of bovine mastitis, is a downstream signaling molecule of TLRs and other immunologic receptors. Activation of the classical TLR4/NF-*κ*B pathway induced by LPS leads to upregulation of proinflammatory cytokines such as interleukin- (IL-) 1*β*, tumor necrosis factor-*α* (TNF-*α*), and IL-6; these inflammatory mediators are then involved in cellular homeostasis and have systemic effects [6, 7]. The inflammatory response has been considered to be a defense response that maintain and defend homeostasis and can accompany many cellular biological processes. It is a complicated process consisting of many parts and their interactions; understanding homeostatic and inflammatory control mechanisms may help to provide an effective therapeutic approach for the conditions associated with inflammatory response.

Recent studies demonstrated that plenty of cell processes such as stress responses, inflammation, and signal transduction rely on the posttranslational functions of ubiquitin and ubiquitin-like proteins (Ubls) [8]. UFM1 modification (ufmylation) was recently proven to be a new posttranslational modification, which has an important effect on lots of pathological and physiological cellular processes [9, 10]. UFL1 (also known as KIAA0776, NLBP, and Maxer), an UFM1 E3 ligase, has been recently identified as a significant regulator of many signaling pathways, including protein ufmylation, unfolded protein response (UPR), and NF-*κ*B signaling regulation [11–14]. Zhang et al. [15] showed that knockdown of UFL1 caused heightened endoplasmic reticulum (ER) stress and activation of the UPR *in vitro* and *in vivo*, whereas UFL1 knockdown caused elevated NF-*κ*B activity. UFL1 can also act as a vital regulator of cellular stress response. Depletion of UFL1 impairs autophagic degradation, increases mitochondrial mass and reactive oxygen species (ROS) production, and leads to the DNA damage response, furthermore, increased the HSC cell death. In addition, UFL1 was reported to possess the ability to bind to LZAP, a tumor suppressor that regulates cell proliferation and inhibits cancer cell invasion [16, 17]. Until now, the biological roles of UFL1 in the inflammatory response of BMECs have not been established. To address this question, we will describe the current and emerging roles of UFL1, with a focus on apoptosis, ER stress, autophagy, oxidative stress, and the TLR4/NF-*κ*B signaling pathway in the regulation of LPS-induced inflammation and cell damage.

## 2. Materials and Methods

### 2.1. Animals and Treatments

Six lactating Holstein cows with (*n* = 3) and without (*n* = 3) mastitis were used in this study. Pathological changes indicating clinical mastitis were signs of udder inflammation, including pain, redness, change of color, and visibly abnormal milk containing flakes. All experiments were carried out in accordance with the National Institutes of Health, and all experimental procedures were approved by the animal care committee of Nanjing Agricultural University.

### 2.2. Histology and Immunohistochemistry Evaluation

To prepare slides for immunohistochemistry staining and histology evaluation, the fresh mammary gland tissue samples were fixed in 4% paraformaldehyde and tissue sections were subsequently dehydrated through steps of graded alcohol, cleared in xylene, and imbedded in paraffin blocks. Sections of 6 *μ*m thickness were cut from each block.

For histological evaluation, the deparaffinised sections were stained with haematoxylin and eosin. Mammary gland sections were then analyzed under a microscope for evidence of injury. For immunohistochemical analysis, the sections were washed by 0.01 M PBS for three times, 3% H_2_O_2_ was added for 30 min to eliminate endogenous peroxidase, washed by PBS for three times again, then the sections were incubated for 1 h in blocking solution (0.3% Triton X-100 and 5% bovine serum albumin (BSA) in PBS), followed by being incubated overnight with UFL1 polyclonal antibody (1 : 400, Proteintech, Chicago, USA) for the detection of UFL1 and then for 1 h with biotinylated goat anti-rabbit IgG secondary antibody. Immunoreactivity was visualized by incubation in DAB. Control staining was performed without the primary antibodies.

### 2.3. Cell Culture, Transfection, and Treatments

Bovine mammary epithelial cells (MAC-T) were a gift from Dr. Youping Sun (Harvard University). Cells were grown to 80–90% confluence in basal media (DMEM media plus 5 mmol/L sodium acetate, 5 mmol/L l-glutamine (GlutaMAX; Invitrogen, Carlsbad, CA, USA), and 20 IU/mL penicillin and streptomycin (Invitrogen, Carlsbad, CA, USA)) supplemented with 10% fetal calf serum (FBS; Sigma-Aldrich, St. Louis, MO, USA). Cells were cultured at 37°C in an atmosphere of 90% humidity and 5% CO_2_. The medium was changed every 48 h. Cells were treated for 12 h with 1 *μ*g/mL LPS (*E. coli* serotype O55:B5, Sigma-Aldrich), and the treatments were performed in basal media without serum.

The UFL1 plasmid was constructed by GenePharma (Shanghai, China) and was identified by JinsiruiBio Company (Nanjing, China). The amplified products were purified and cloned into the pEX-3 vector. MAC-T cells were cultured to a confluency of 70-80% in 6-well dishes and transfected with 2 *μ*g of the pcDNA3.1-UFL1 or the pcDNA3.1 empty vector in Opti-MEM (Gibco, Carlsbad, CA, USA) using Lipofectamine 2000 (Invitrogen, Carlsbad, CA, USA) for 6 h following the manufacturer's specifications. Then, 48 h after transfection, the cells were treated as indicated above.

MAC-T cells were transfected with UFL1 and TLR4 siRNA and negative control siRNA (GenePharma, Shanghai, China) using Lipofectamine 2000 (Invitrogen, Carlsbad, CA, USA) according to the manufacturer's instructions. Then, 48 h after transfection, the cells were treated as indicated above. The UFL1 siRNA sequence is 5′-GCAGCAGAAGCUUGUGAUATT-3′, and the antisense sequence is 5′-UAUCACAAGCUUCUGCUGCTT-3′. The TLR4 siRNA sequence is 5′-GGACCUCUCUAAGUGUCAATT-3′, and the antisense sequence is UUGACACUUAGAGAGGUCCTT.

### 2.4. Assay for Malondialdehyde, Superoxide Dismutase, and Catalase

BMECs (5 × 10^3^ cells/well in 96-well plates) were treated as described above. Then, the cellular superoxide dismutase (SOD), catalase (CAT), and malondialdehyde (MDA) were measured with corresponding assay kits (Nanjing Jiancheng Bioengineering Institute, Nanjing, China) according to the manufacturer's protocol. The optical density of MDA, SOD, and CAT was measured at absorption wavelengths of 532 nm, 450 nm, and 405 nm, respectively, with a microplate reader. Levels of SOD, CAT, and MDA were calculated and normalized to the normal control.

### 2.5. Flow Cytometer Detection of Apoptosis

An Annexin V-FITC/PI Detection Kit (BD Biosciences, San Diego, CA, USA) was used for the determination of cell apoptosis. The cells were transfected as described above and then stimulated with LPS for 12 h. Following the manufacturer's instructions, cells were stained with Annexin V-FITC and PI at room temperature for 25 min. Then, apoptosis was measured with a FACSCalibur (BD Biosciences, Bedford, MA, USA) flow cytometer (FCM). The data analysis was performed using the FlowJo software.

### 2.6. Caspase-3 Activity Measurement

BMEC protein lysates were centrifuged at 18000 rpm for 15 min at 4°C, and supernatants were quantified for caspase-3 enzymatic activity using a Caspase Apoptosis Assay Kit (Geno Technology Inc., St. Louis, MO, USA) according to the manufacturer's instructions.

### 2.7. Immunofluorescent Staining

Cells were fixed, permeabilized, and blocked. They were then incubated overnight at 4°C with primary antibodies: rabbit anti-UFL1 (1 : 200, Proteintech) and rabbit anti-NF-*κ*B P65 (1 : 200, Cell Signaling). After incubating overnight with primary antibodies, secondary fluorescent antibodies, Alexa 488-conjugated anti-rabbit Ab (1 : 400), or Alexa 568-conjugated anti-mouse Ab (1 : 400) were added for 1 h and DAPI was used for nuclear counterstaining. The images were photographed using a fluorescence microscope (Olympus, Tokyo, Japan), and the fluorescence intensity was assessed using the ImageJ software (National Institutes of Health, Bethesda, MD, USA).

### 2.8. Real-Time Quantitative PCR Analysis

Total RNA was extracted using the TRIzol reagent (Invitrogen, Carlsbad, CA, USA), and cDNA was synthesized using a PrimeScript™ RT Master Mix (TaKaRa, Japan) according to the manufacturer's protocols. Real-time quantitative PCR was performed using the standard protocols on an Applied Biosystems 7500 HT Sequence detection system using SYBR® Premix Ex Taq™ (TaKaRa, Japan). The following primers were used for qPCR analysis: UFL1, forward, 5′-TGTGGATCAGGTGGAAGCAT-3′ and reverse, 5′-TACAGCTGAAGCCTGTTTGC-3′; *TNF-α*, forward, 5′-CAAGTAACAAGCCGGTAGCC-3′ and reverse, 5′-CCCTGAAGAGGACCTGTGAG-3′; *IL-6*, forward, 5′-TGAGTGTGAAAGCAGCAAGG-3′ and reverse, 5′-AAGACCAGCAGTGGTTCTGA-3′; *IL-1β*, forward, 5′-AGTGCAAACTCCAGGACAGA-3′ and reverse, 5′-GATACCCAAGGCCACAGGAA-3′; and Bos taurus *GAPDH*, forward, 5′-CATGACCACTTTGGCATCGT-3′ and reverse, 5′-CCATCCACAGTCTTCTGGGT-3′. Gene expression data were normalized to that of glyceraldehyde-3-phosphate dehydrogenase (GAPDH) by employing an optimized comparative Ct (2^-ΔΔCt^) value method.

### 2.9. Enzyme-Linked Immunosorbent Assay (ELISA)

The BMECs were transfected as described above and then stimulated with LPS for 12 h. The supernatant levels of TNF-*α*, IL-6, and IL-1*β* were measured using a commercially available enzyme-linked immunosorbent assay kit (R&D Systems, Shanghai, China) according to the manufacturer's instructions. The results were expressed as pg/mg protein.

### 2.10. Western Blot Analysis

Whole BMEC protein lysates were centrifuged at 13000 rpm for 15 min at 4°C. Nuclear extracts were prepared using the NE-PER nuclear and cytoplasmic extraction reagents (Thermo Scientific) according to the instructions of the manufacturer. Protein concentration was quantified in the supernatants using a BCA protein assay kit (Beyotime, China). Proteins were separated by SDS-PAGE and transferred to a PVDF membrane. The membranes were probed with the following primary antibodies: rabbit anti-UFL1 (1 : 1000, Proteintech), rabbit anti-Bax (1 : 1000, Proteintech), rabbit anti-Bcl2 (1 : 1000, Wanleibio), rabbit anti-HSP70 (1 : 2000, Cell Signaling), rabbit anti-GRP78 (1 : 1000, Proteintech), rabbit anti-CHOP (1 : 1000, LifeSpan), rabbit anti-LC3B (1 : 2000, Novus), rabbit anti-P62 (1 : 1000, Sigma), rabbit anti-TLR4 (1 : 1000, Bioss), rabbit anti-NF-*κ*B P65 (1 : 1000, Cell Signaling), rabbit anti-phospho-NF-*κ*B P65 (1 : 1000, Cell Signaling), rabbit anti-I*κ*B*α* (1 : 1000, Cell Signaling), rabbit anti-phospho-I*κ*B*α* (1 : 1000, Cell Signaling), and rabbit GAPDH (1 : 5000, Proteintech). The blots were incubated with HRP-conjugated secondary antibodies, and the signals were detected by enhanced chemiluminescence (ECL) Western blot detection reagents (Pierce, Rockford, IL, USA). Immunoblots were scanned, and densitometry was performed using the ImageJ software (National Institutes of Health, Bethesda, MD, USA).

### 2.11. Statistical Analysis

Statistical analyses were performed using the GraphPad Prism 6.01 software (GraphPad Software Inc., San Diego, CA). All data are shown as the mean ± SEM as indicated. The significance of a difference between different treatments was determined by one-way ANOVA analysis. *p* < 0.05 was considered a significant difference.

## 3. Results

### 3.1. LPS Increases UFL1 Expression in Bovine Mammary Gland Tissue and BMECs

**Figure 1:**
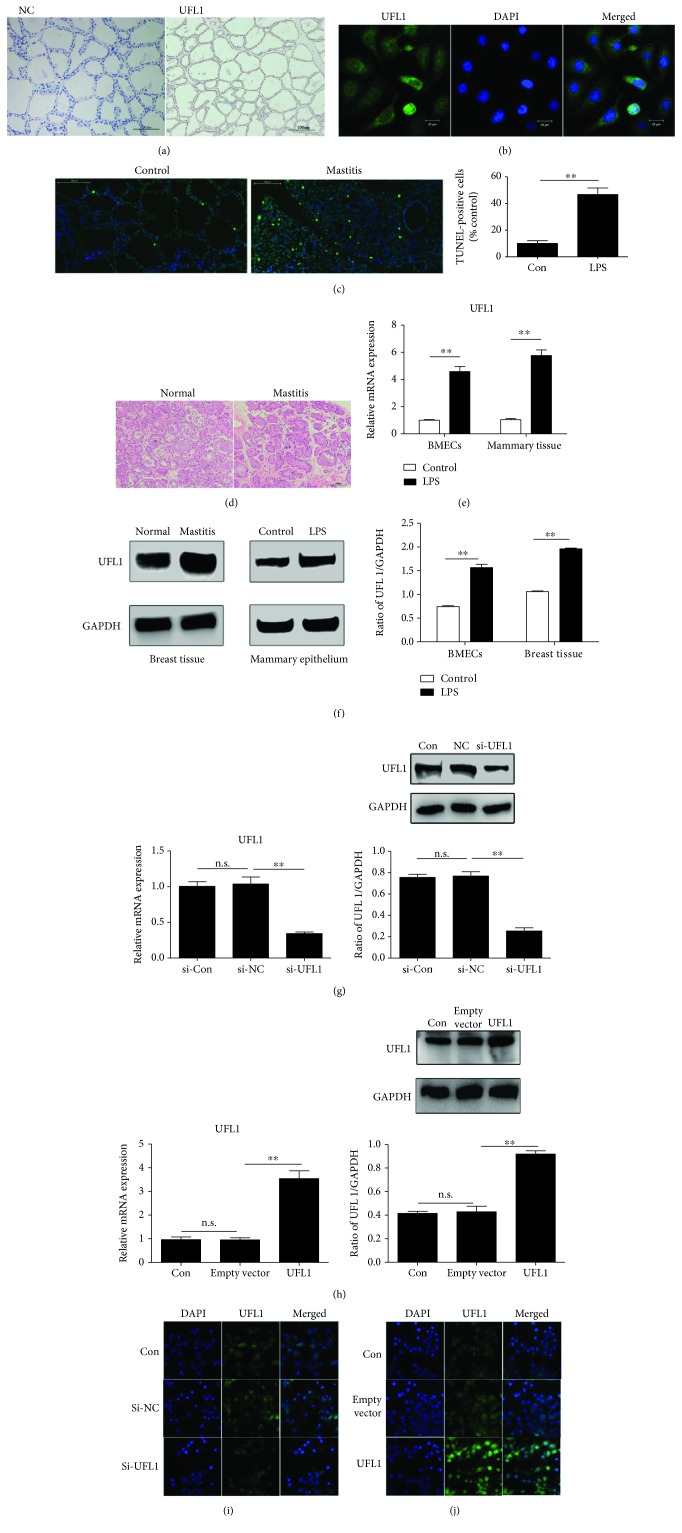
UFL1 is highly expressed in LPS-induced bovine mammary gland tissue and BMECs. (a) Bovine mammary glands were analyzed by immunohistochemistry. (b) Immunofluorescent staining of BMECs with anti-UFL1 antibody. (c) Representative TUNEL staining of normal and mastitis bovine mammary gland. (d) Representative H&E staining of normal and mastitis bovine mammary gland. (e) Expression of UFL1mRNA in bovine mammary gland tissue and BMECs treated with LPS. (f) Expression of UFL1 protein in bovine mammary gland tissue and BMECs treated with LPS. (g) Effect of UFL1 siRNA on the expression of UFL1 in BMECs. (h) UFL1 expression after BMECs was transfected with UFL1 overexpression plasmid. (i) Immunofluorescent staining of BMECs with anti-UFL1 antibody in siRNA-treated cells stimulated with LPS. (j) Immunofluorescent staining of BMECs with anti-UFL1 antibody in UFL1 overexpression plasmid-treated cells stimulated with LPS. Data are presented as the means ± the standard errors of the mean (SEM) of three independent experiments. ^∗∗^*p* < 0.01.

UFL1 has recently been identified as an important regulator of the cellular stress response, but no studies have addressed the effect of UFL1 on BMECs, and the role of UFL1 in bovine mastitis is still unknown. To determine whether UFL1 is involved in the LPS-induced inflammatory response in bovine mammary gland tissue and BMECs, we first evaluated the cellular localization of UFL1. Bovine mammary glands were analyzed by immunohistochemistry, and as shown in Figure 1(a), UFL1 was expressed in epithelial cells of the bovine mammary gland. In addition, immunostaining of BMECs with anti-UFL1 antibody revealed that UFL1 was expressed in both the cytoplasm and the nucleus (Figure 1(b)). Then, we analyzed the expression levels of UFL1 in LPS-induced bovine mammary gland tissue and BMECs using real-time PCR and Western blotting. Notably, we observed that the mRNA (Figure 1(e)) and protein (Figure 1(f)) expression levels of UFL1 in bovine mammary gland tissue and BMECs were significantly increased after LPS treatment. Based on these results, we hypothesized that UFL1 could play a role in the LPS-induced inflammatory response. To further investigate the possible involvement of UFL1 in bovine mastitis, we downregulated UFL1 expression in BMECs using small interfering RNA, and the results showed that UFL1 siRNA could suppress the expression of UFL1 mRNA and protein (Figures 1(g) and 1(i)). Additional support for the idea that UFL1 is involved in bovine mastitis was provided by experiments in which UFL1 was overexpressed. As shown in Figures 1(h) and 1(j), UFL1 was overexpressed after BMECs were transfected with an UFL1 overexpression plasmid.

### 3.2. UFL1 Modulates Apoptosis in LPS-Stimulated BMECs

**Figure 2:**
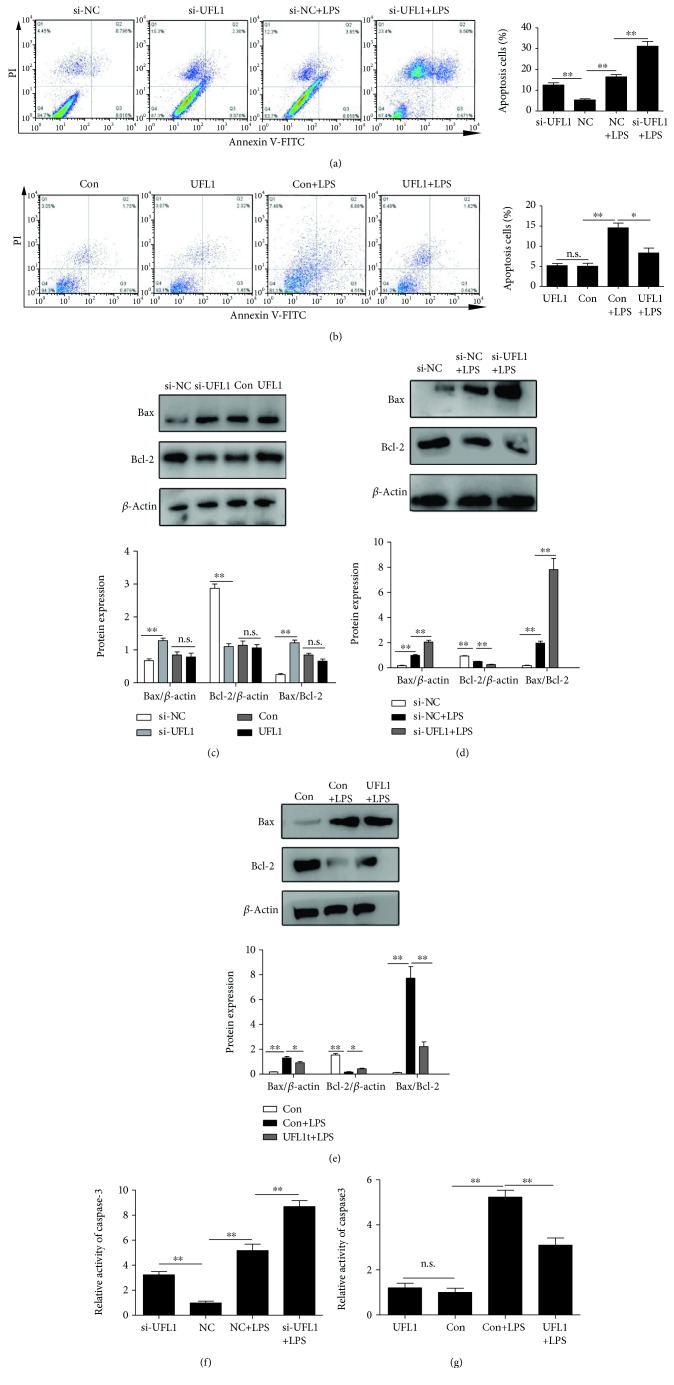
UFL1 protects the BMECs against LPS-induced inflammatory damage. (a) Effect of UFL1 siRNA on apoptosis in LPS-challenged BMECs. (b) Effect of UFL1 overexpression on apoptosis in LPS-challenged BMECs. (c) Effect of UFL1 on the protein expression of Bax and BCl-2 in BMECs. (d) Representative Western blots and quantitative evaluation of Bax and Bcl-2 in UFL1 siRNA-transfected BMECs stimulated with LPS. (e) Representative Western blots and quantitative evaluation of Bax and Bcl-2 in BMECs transfected with UFL1 overexpression plasmid stimulated with LPS. (f) Effect of UFL1 siRNA on activity of caspase-3 in LPS-challenged BMECs. (g) Effect of UFL1 overexpression on activity of caspase-3 in LPS-challenged BMECs. Data are presented as the means ± the standard errors of the mean (SEM) of three independent experiments. ^∗^*p* < 0.05; ^∗∗^*p* < 0.01.

To examine the effects of UFL1 on LPS-stimulated BMECs, we first evaluated the potential role of UFL1 in regulating cell apoptosis following LPS treatment. The apoptosis of BMECs was analyzed via double-labelled flow cytometry using Annexin V-FITC/PI. As shown in Figure 2(a), LPS treatment and UFL1 silencing effectively increased cell apoptosis and knockdown of UFL1 resulted in a significant further increase in apoptosis in LPS-challenged BMECs. Conversely, compared with the Con+LPS group, UFL1-overexpressing cells had a much lower number of apoptotic cells (Figure 2(b)). Consistent with the apoptosis results as detected by flow cytometry, the protein expression of Bax/Bcl-2 and activity of caspase-3, the well-known apoptosis markers, were significantly increased in UFL1-depleted cells in response to LPS (Figures 2(c), 2(d), and 2(f)) and were markedly attenuated in LPS-stimulated UFL1-overexpressing cells compared with those in the Con+LPS group (Figures 2(e) and 2(g)). Taken together, these data revealed that UFL1 could play a role in the LPS-induced apoptosis. Overexpression of UFL1 could protect against apoptosis in LPS-stimulated BMECs.

### 3.3. UFL1 Modulates ER Stress in LPS-Stimulated BMECs

**Figure 3:**
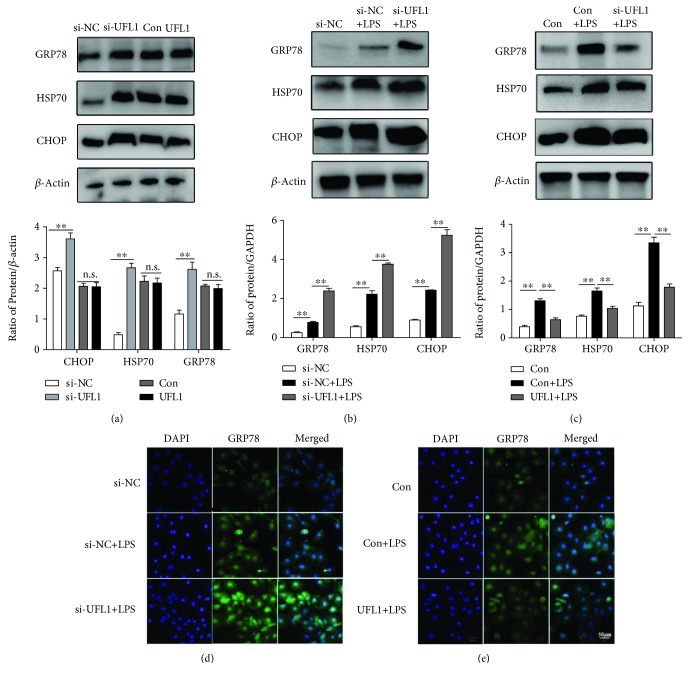
UFL1 mediates ER stress in LPS-stimulated BMECs. (a) Effect of UFL1 on the protein expression of CHOP, GRP78, and HSP70 in BMECs. (b) Representative Western blots and quantitative evaluation of CHOP, GRP78, and HSP70 in UFL1 siRNA-transfected BMECs stimulated with LPS. (c) Representative Western blots and quantitative evaluation of CHOP, GRP78, and HSP70 in BMECs transfected with UFL1 overexpression plasmid stimulated with LPS. (d) Immunofluorescent staining of BMECs with anti-GRP78 antibody in siRNA-treated cells stimulated with LPS. (e) Immunofluorescent staining of BMECs with anti-GRP78 antibody in UFL1 overexpression plasmid-treated cells stimulated with LPS. Data are presented as the means ± the standard errors of the mean (SEM) of three independent experiments. ^∗∗^*p* < 0.01.

Recently, substantial evidence has certified that inflammation is connected with endoplasmic reticulum (ER) stress; activation of the NF-*κ*B signaling pathway and proinflammatory cytokines could trigger ER stress. Sustained ER stress could exacerbate the inflammatory response process and cellular damage [18, 19]. To determine whether LPS induced ER stress in mammary epithelial cells, we assessed the expression of ER stress markers. As shown in Figure 3, LPS treatment and UFL1 silencing significantly induced the expression of CHOP, a proapoptotic transcription factor that is overexpressed following the disruption of ER homeostasis, and increased the expression of Hsp70, which is normally upregulated during ER stress. Moreover, GRP78, a member of the HSP protein family found in the ER that is generally upregulated after ER stress, was also increased. Then, we examined the effects of UFL1 on LPS-induced ER stress, and we found that UFL1 siRNA-mediated knockdown strongly enhanced the effects of LPS on ER stress in LPS-challenged BMECs, as indicated by the increased expression of CHOP, Hsp70, and GRP78 (Figures 3(b) and 3(d)). However, UFL1-overexpressing cells showed a marked decrease in the levels of CHOP, GRP78, and HSP70 compared with those in the Con+LPS group following LPS challenge (Figures 3(c) and 3(e)). These data indicate that ER stress is activated during the development of the LPS-induced inflammatory response; moreover, notably, UFL1 can mediate LPS-induced ER stress, and overexpression of UFL1 can relieve the LPS-induced ER stress in mammary epithelial cells.

### 3.4. UFL1 Modulates Autophagy in LPS-Stimulated BMECs

**Figure 4:**
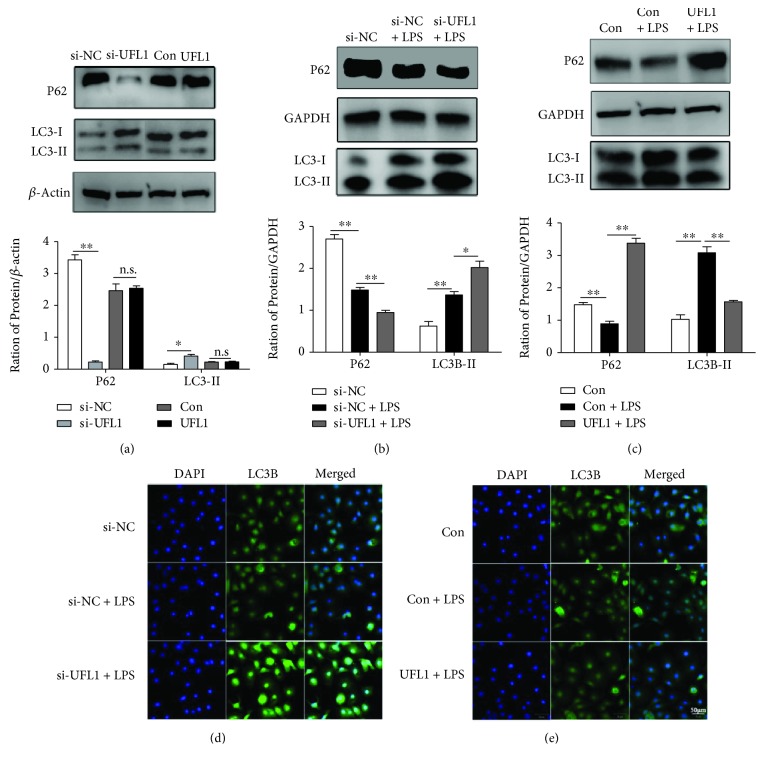
UFL1 mediates autophagy in LPS-stimulated BMECs. (a) Effect of UFL1 on the protein expression of P62 and LC3-II in BMECs. (b) Representative Western blots and quantitative evaluation of P62 and LC3-II in UFL1 siRNA-transfected BMECs stimulated with LPS. (c) Representative Western blots and quantitative evaluation of P62 and LC3-II in BMECs transfected with UFL1 overexpression plasmid stimulated with LPS. (d) Immunofluorescent staining of BMECs with anti-LC3B antibody in siRNA-treated cells stimulated with LPS. (e) Immunofluorescent staining of BMECs with anti-LC3B antibody in UFL1 overexpression plasmid-treated cells stimulated with LPS. Data are presented as the means ± the standard errors of the mean (SEM) of three independent experiments. ^∗^*p* < 0.05; ^∗∗^*p* < 0.01.

ER stress is strictly connected to autophagy, and growing evidence has linked defective autophagy to the pathogenesis and progression of inflammatory injury [20]. Thus, we studied the autophagy process and further investigated the effect of UFL1 on autophagy in LPS-stimulated BMECs. As shown in Figures 4(a), 4(b), and 4(c), LPS treatment and UFL1 silencing robustly augmented the expression of LC3-II, which is an essential protein in the process of autophagy and now widely used to monitor this process. The effect of LPS on autophagy activation was also confirmed by the decreased expression of P62, a protein degraded during the autophagy process (Figures 4(a), 4(b), and 4(c)). Meanwhile, UFL1 depletion considerably promoted the excessive activation of autophagy in LPS-stimulated BMECs (Figures 4(b) and 4(d)). Nevertheless, UFL1 overexpression blocked the effect of LPS and significantly restored the expression of autophagy proteins (Figures 4(c) and 4(e)). These observations indicate that UFL1 overexpression can relieve LPS-induced abnormal autophagy in mammary epithelial cells and that the reduced autophagy likely contributes to the functional consequences of the inflammatory process.

### 3.5. UFL1 Modulates Oxidative Stress in LPS-Stimulated BMECs

**Figure 5:**
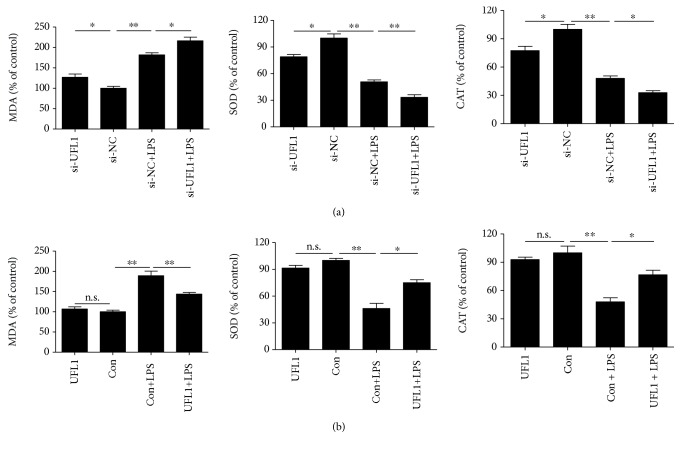
UFL1 mediates oxidative stress in LPS-stimulated BMECs. (a) Effect of UFL1 siRNA on the levels of MDA, SOD, and CAT in LPS-challenged BMECs. (b) Effect of UFL1 overexpression on the levels of MDA, SOD, and CAT in LPS-challenged BMECs. Data are presented as the means ± the standard errors of the mean (SEM) of three independent experiments. ^∗^*p* < 0.05; ^∗∗^*p* < 0.01.

Oxidative stress, which has been shown to be largely involved in several pathological processes *in vitro*, exhibits a considerable cross talk between ER stress, apoptosis, autophagy, and inflammatory cytokines [21–23]. In addition, UFL1 has been proven to be closely associated with the ROS production; loss of UFL1 can lead to accumulation of ROS [15]. We therefore assessed the roles of UFL1 in modulating oxidative stress. As shown in Figure 5, compared with the control group, LPS treatment and UFL1 silencing significantly decreased the levels of SOD and CAT and the levels of MDA have a significant increase. Moreover, we found that UFL1 siRNA-mediated knockdown strongly enhanced the effects of LPS on oxidative stress in LPS-challenged BMECs, as indicated by the decreased levels of SOD and CAT and the increased level of MDA (Figure 5(a)). However, UFL1-overexpressing cells showed a marked increase in the levels of SOD and CAT and a decreased level of MDA, when compared with those in the Con+LPS group following LPS challenge (Figure 5(b)). These data indicate that oxidative stress is activated in BMECs during the LPS treatment; moreover, overexpression of UFL1 could relieve the LPS-induced oxidative stress in bovine mammary epithelial cells.

### 3.6. UFL1 Modulates the TLR4/NF-*κ*B Signaling Pathway in LPS-Stimulated BMECs

**Figure 6:**
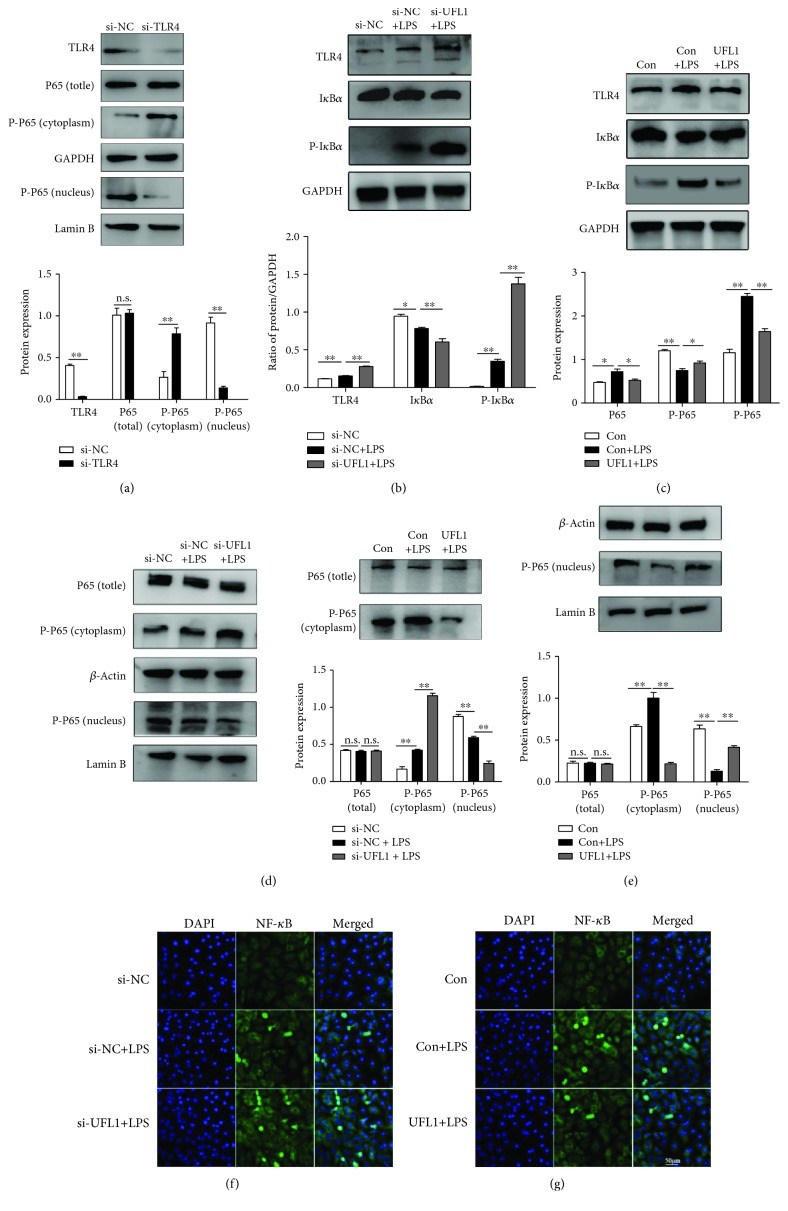
UFL1 modulates the TLR4/NF-*κ*B signaling pathway in LPS-stimulated BMECs. (a) The effects of si-TLR4 transfection on TLR4 and NF-*κ*B P65 expression. (b) Representative immunoblots and quantification for analysis of TLR4, I*κ*B*α*, and phospho-I*κ*B*α* in UFL1 siRNA-transfected BMECs stimulated with LPS. (c) Representative immunoblots and quantification for analysis of TLR4, I*κ*B*α*, and phospho-I*κ*B*α* in BMECs transfected with UFL1 overexpression plasmid. (d) The expression of p65 and p-p65 in the cytoplasm and nucleus of UFL1 siRNA-transfected BMECs stimulated with LPS. (e) The expression of p65 and p-p65 in the cytoplasm and nucleus of BMECs transfected with UFL1 overexpression plasmid. (f) Immunofluorescent staining of BMECs with anti-NF-*κ*B antibody in siRNA-treated cells stimulated with LPS. (g) Immunofluorescent staining of BMECs with anti-NF-*κ*B antibody in UFL1 overexpression plasmid-treated cells stimulated with LPS. Data are presented as the means ± the standard errors of the mean (SEM) of three independent experiments. ^∗^*p* < 0.05; ^∗∗^*p* < 0.01.

During infection, inflammatory cells are recruited to the site of damage, provoking the production of numerous cytokines by local cells expressing TLRs. NF-*κ*B is an important transcription factor located downstream of the TLR4-mediated signaling pathway and plays an essential role in regulating the immune response. In the present study, we first knocked down TLR4 by siRNA to confirm that the activation of NF-*κ*B is mediated by TLR4 in LPS-stimulated BMECs. As determined by immunoblotting assay, after transfection with si-TLR4, the expression of TLR4 and subsequent expression of NF-*κ*B p65 in the nuclear were both muted (Figure 6(a)). Furthermore, UFL1 depletion significantly increased the expression of TLR4, phospho-NF-*κ*B P65, and phospho-I*κ*B*α* (Figures 6(b) and 6(d)), whereas overexpression of UFL1 led to a marked reduction in the expression of TLR4, phospho-NF-*κ*B P65, and phospho-I*κ*B*α* after LPS treatment (Figures 6(c) and 6(e)). No significant difference was observed in the NF-*κ*B P65 levels between the overexpression and knockdown groups under LPS treatment (Figures 6(d) and 6(e)). As expected, immunofluorescence analysis of BMECs with NF-*κ*B P65 antibody also indicated that LPS induced more activation of NF-*κ*B in UFL1-silenced cells (Figure 6(f)) and overexpression UFL1 suppressed the activation of NF-*κ*B (Figure 6(g)). These results indicate that UFL1 depletion enhances the activation of the LPS-induced TLR4/NF-*κ*B signaling pathway and that UFL1 overexpression can suppress the activation of the TLR4/NF-*κ*B pathway, suggesting that UFL1 can control the inflammatory response by inhibiting NF-*κ*B activation.

### 3.7. UFL1 Modulates Cytokine Expression in LPS-Stimulated BMECs

**Figure 7:**
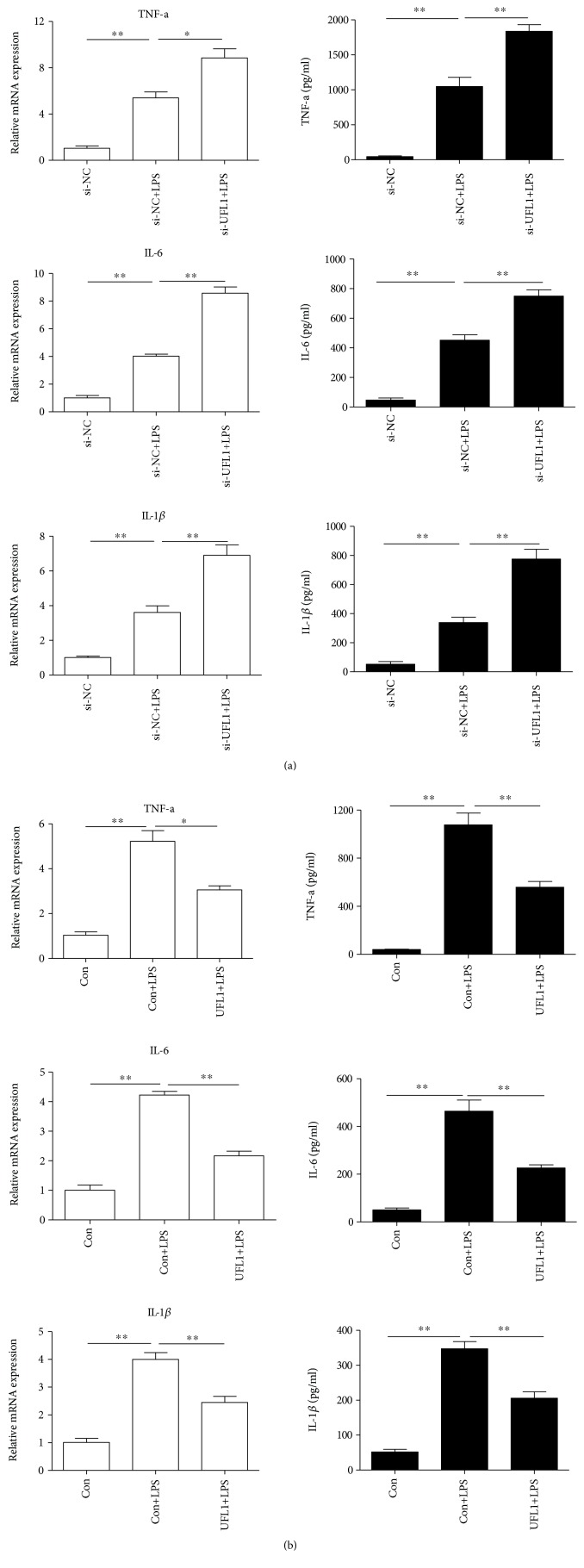
UFL1 modulates cytokine expression in LPS-stimulated BMECs. (a) qPCR and ELISA analysis of TNF-*α*, IL-6, and IL-1*β* in UFL1 siRNA-transfected BMECs stimulated with LPS. (b) qPCR and ELISA analysis of TNF-*α*, IL-6, and IL-1*β* in BMECs transfected with UFL1 overexpression plasmid stimulated with LPS. Data are presented as the means ± the standard errors of the mean (SEM) of three independent experiments. ^∗^*p* < 0.05; ^∗∗^*p* < 0.01.

Since proinflammatory cytokines are the downstream targets of NF-*κ*B-mediated transcriptional regulation and primarily involved in immune and inflammatory responses, we then examined the role of UFL1 in proinflammatory cytokine production. As shown in Figure 7(a), UFL1-silenced cells exhibited higher levels of TNF-*α*, IL-6, and IL-1*β* after LPS treatment compared to those in the NC+LPS group (Figure 7(a)). On the contrary, overexpression of UFL1 inhibited the expression of LPS-induced proinflammatory cytokines (Figure 7(b)). These observations indicate that UFL1 can modulate the TLR4/NF-*κ*B signaling pathway in LPS-stimulated BMECs, then inhibit the LPS-induced expression of proinflammatory cytokines, and modulate ER stress and autophagy, thereby providing therapeutic interventions for diseases associated with inflammation, including bovine mastitis.

## 4. Discussion

Our study focused on the biological roles of UFL1 in the LPS-induced inflammatory response of BMECs. The experimental results here demonstrated that UFL1 depletion aggravated the LPS-induced inflammatory response and cell damage by positively regulating the TLR4/NF-*κ*B pathway, exacerbating LPS-induced ER stress, apoptosis, autophagy, and oxidative stress. Overexpression of UFL1 suppressed the activation of the TLR4/NF-*κ*B pathway and reduced the LPS-induced ER stress, apoptosis, autophagy, and oxidative stress, thereby alleviating the inflammatory response and cell damage. These findings indicate that UFL1 represents a novel regulator of the inflammatory response and provide a possible explanation for how UFL1 plays an important role in bovine mastitis.

The mammary epithelium is activated rapidly in response to bacterial infection and exerts diverse actions. Our study found that a significant increase in cell apoptosis was induced by LPS in BMECs (Figure 2), which was consistent with the research results obtained previously [24, 25]. Thus, we confirmed that LPS invasion could injure BMECs. UFL1, the most recently identified protein, can act as a key regulator of the cellular stress response, yet its physiological function in LPS-stimulated bovine mammary epithelial cells (BMECs) remains unknown. In the present study, UFL1 silencing increased cell apoptosis; these results are consistent with those obtained by Liu et al. [25] and Lemaire et al. [26]. In addition, we showed that UFL1 was highly expressed in LPS-induced bovine mammary gland tissue and BMECs. Overexpression of UFL1 protected against apoptosis in LPS-stimulated BMECs. These findings suggest that UFL1 can regulate functions independently of its E3 ligase activity in ufmylation.

It has been well established that UFL1 is mainly located in the endoplasmic reticulum (ER) and may be involved in ER stress-associated cellular processes and homeostasis [27]. Loss of UFL1 leads to elevated ER stress and activation of the UPR *in vitro* and *in vivo* [15, 26]. In our present study, LPS caused ER stress in the mammary epithelium, as indicated by the increased expression of CHOP, Hsp70, and the molecular chaperon GRP78 (Figure 3). Notably, silencing UFL1 resulted in a marked increase in the expression of these ER stress markers (Figures 3(a), 3(b), and 3(d)), and UFL1 overexpression relieved the LPS-induced ER stress in the mammary epithelial cells (Figures 3(a), 3(c), and 3(e)). These results are consistent with the previous study on the cytoprotective role of the Ufm1 system in ER stress-induced apoptosis of pancreatic *β*-cells. Collectively, our data combined with recent findings indicate that there exists a tight link between ER stress and inflammation in LPS-stimulated BMECs, suggesting that a therapeutic strategy that targets the common molecular processes that are altered in stressed BMECs might be effective. UFL1 provides such a target.

Another important finding of the present study is the role of UFL1 in the modulation of autophagy in mammary epithelium after LPS administration. Autophagy is in a dynamic equilibrium in the cell, and its impairment can lead to metabolic dysfunctions and cell death. Additionally, autophagy is also an essential, homeostatic process affecting immunity [28, 29]; the autophagy pathway and proteins are found to balance the advantageous and baneful effects of immunity and inflammation and thereby may protect against infection and inflammatory responses [30]. In this study, we found that LC3-II protein expression was particularly higher and P62 was markedly lower in UFL1-depleted cells in response to LPS treatment and UFL1 silencing (Figures 4(a), 4(b), and 4(d)), indicating that a lack of UFL1 aggravates LPS-induced autophagy. Conversely, UFL1 overexpression significantly alleviated LPS-induced abnormal autophagy in stimulated BMECs, as indicated by the decrease in LC3 II expression and the increased P62 protein level (Figures 4(c) and 4(e)). Our results are consistent with the previous research by Levine et al., which showed that P62 as well as LC3 could act as cellular adaptor proteins to coordinate innate immune responses [30]. Growing lines of evidence link defective autophagy to the pathogenesis and progression of several inflammatory injuries [31, 32]. Excessively activated autophagy may release some damage-associated molecular pattern molecules (DAMPs), including ATP and ROS, which activate the inflammatory process. Moreover, the reduced autophagy may likely contribute to the functional consequences of the inflammatory process. Taken together, our results revealed an important role of UFL1 in maintaining the autophagy process during adverse conditions.

Oxidative response, ER stress, apoptosis, autophagy, and inflammation constitute the major defense networks that let cells to get used to and survive stress conditions imposed by lots of pathological and physiological stimuli. Oxidative stress is part of the immune response to harmful pathogens, such as LPS, on the cell wall of gram-negative bacteria [33]. In our study, UFL1 overexpression exhibited efficacy for inhibiting MDA and increasing SOD and CAT levels induced by LPS (Figure 5(b)), suggesting an antioxidative effect of UFL1. Studies in various organisms showed that oxidative stress was invoked by ROS overproduction, thus, the antioxidative effect of UFL1 may be related to its regulation of ROS production [15].

The cellular biological processes described above, such as apoptosis, ER stress, autophagy, and oxidative stress, are regulated by processes involving common transcription factors and signaling pathways including NF-*κ*B [34, 35]. Strategies aimed at modulating these cellular biological processes may lead to therapeutic interventions for diseases associated with inflammation, including bovine mastitis.

The inflammatory response of BMECs is initiated following the detection of pathogens via specialized PRRs, e.g., toll-like receptors (TLRs) and nucleotide oligomerization domain-like receptors (NLRs) [35]. NF-*κ*B, which is a central player in TLR- and NLR-mediated inflammation, is involved in the inflammatory processes associated with the development of bovine mastitis [36]. NF-*κ*B activation strongly enhances the phosphorylation and proteasomal degradation of the I*κ*B proteins, leading to the release and phosphorylation nuclear factor *κ*B dimmers, which are able to enter the nucleus and trigger the expression of inflammatory cytokines [37–40]. Consequently, inhibition of NF-*κ*B activation has attracted increased attention as a therapeutic approach for intervention in immune and inflammatory events [41]. Previous studies have demonstrated that UFL1 inhibits NF-*κ*B activity and NF-*κ*B-mediated transcription [12, 16]; thus, we hypothesized that UFL1 might be able to control the inflammatory response by inhibition of NF-*κ*B activation. In the present study, our data showed that the expression of TLR4, the degradation of I*κ*B*α*, and the phosphorylation of p65 were significantly increased after stimulation with LPS (Figures 5(a) and 5(b)). UFL1 depletion significantly increased the expression of TLR4, degradation of I*κ*B*α*, and the phosphorylation of p65, subsequently leading to higher levels of proinflammatory cytokines (Figures 5(a), 5(c), and 6(a)). Conversely, overexpression of UFL1 suppressed the expression of TLR4, the degradation of I*κ*B*α*, and the phosphorylation of p65, thereby inhibiting the LPS-induced expression of proinflammatory cytokines (Figures 5(b), 5(d), and 6(b)). These results provide convincing evidence that UFL1 plays an inhibitory role in inflammatory responses by targeting NF-*κ*B nuclear translocation. We further demonstrated that UFL1 interacts with I*κ*B*α* and regulates its stability, thereby regulating NF-*κ*B transcriptional activity and the expression of NF-*κ*B target genes. Our findings identify UFL1 as an important regulator of the TLR4/NF-*κ*B pathway.

Collectively, UFL1 is a protein with many functions. Although it was originally characterized as an UFM1 E3 ligase that plays essential roles in protein ufmylation, its function seems to be more diverse. This study provides convincing evidence that UFL1 can engage the TLR4/NF-*κ*B pathway and efficiently mediates LPS-induced apoptosis, ER stress, autophagy, and oxidative stress, thereby alleviating the inflammatory response and cell damage. The ability of UFL1 to regulate inflammation *in vitro* suggests that UFL1 could be a potential therapeutic target for bovine mastitis.

## Data Availability

No data were used to support this study.
